# Optimizing Sequential Targeted Therapies in Advanced Renal Cell Carcinoma Using Patient-Derived Orthotopic Xenograft Mouse Avatars

**DOI:** 10.3390/cancers18101615

**Published:** 2026-05-16

**Authors:** Amita Bhattarai, Ravan Moret, Xin Zhang, Grace Maresh, Henry Yip, Carl Haupt, Rachel Graham, Maria Latsis, Marc Matrana, Kyle Rose, Stephen Bardot, Li Li

**Affiliations:** 1 Laboratory of Translational Cancer Research, Ochsner Clinic Foundation, 1514 Jefferson Highway, New Orleans, LA 70121, USA; amitabhattarai17@gmail.com (A.B.); poche.moret@gmail.com (R.M.); xzhang@ochsner.org (X.Z.); grace.maresh@ochsner.org (G.M.); henryyip0118@gmail.com (H.Y.); carl.haupt@ochsner.org (C.H.); 2 Urology Department, Ochsner Clinic Foundation, 1514 Jefferson Highway, New Orleans, LA 70121, USA; rgraham@ochsner.org (R.G.); mlatsis@ochsner.org (M.L.); kyle.rose@ochsner.org (K.R.); 3 Department of Hematology and Oncology, Ochsner Clinic Foundation, New Orleans, LA 70121, USA; mamatrana@ochsner.org; 4 Ochsner Clinical School, University of Queensland, New Orleans, LA 70121, USA

**Keywords:** renal cell carcinoma, PDOX, sequential therapy, targeted therapy, sunitinib, pazopanib, everolimus, PD-L1, personalized medicine

## Abstract

**Background/Objectives:** Advanced renal cell carcinoma (aRCC) remains incurable, with no established optimal sequence of targeted therapies due to interpatient heterogeneity and acquired resistance. We developed a luciferase-enabled patient-derived orthotopic xenograft (PDOX) avatar platform to evaluate sequential targeted therapies in individualized aRCC models that recapitulate tumor architecture, proliferation, angiogenesis, metastasis, and PD-L1 expression. **Methods:** Tumor specimens from two renal cell carcinoma (RCC) patients were expanded subcutaneously in NOD/SCID mice, transduced with luciferase/red fluorescent protein (Luc/RFP), and orthotopically implanted into mouse kidneys (KiCa-Pt58: sarcomatoid RCC, pT3aN1M1, Fuhrman grade 4; KiCa-Pt118: clear cell RCC with sarcomatoid component, pT3aNxM0, Fuhrman grade 4, respectively). Tumor growth and metastasis were monitored weekly by bioluminescence imaging (BLI). Mice were randomized into vehicle control or four sequential treatment groups (Everolimus→Sunitinib [E→S], Sunitinib→Everolimus [S→E], Pazopanib→Sunitinib [P→S], Pazopanib→Everolimus [P→E]). Drugs were administered orally three times weekly until resistance (>200% BLI increase), with one switch. At necropsy, tumor burden, ex vivo BLI metastasis, weights, H&E histology, and immunohistochemistry (Ki67, CD44, CD31, PD-L1) were assessed. **Results:** Two independent experiments were performed. In dosing optimization, PDOX tumors recapitulated parental histology and proliferative indices, mirroring patient trajectories. KiCa-Pt58 (metastatic sarcomatoid RCC; deceased 1-month post-nephrectomy) showed aggressive features: rapid engraftment at low doses, early growth (week 2), and lung metastases in 78% of mice (sacrifice day 34), reflecting a fulminant course. KiCa-Pt118 (non-metastatic; patient recurrence-free >8 years post nephrectomy) exhibited indolent behavior: delayed engraftment requiring higher doses plus lymph node stromal (HK) support, slower growth (week 4), no metastases, and later sacrifice (day 78), consistent with remission. In sequential therapy evaluation, for KiCa-Pt58, P→E yielded greatest reductions in tumor weight (*p* < 0.01), lung metastases (*p* < 0.01), Ki67+ proliferation, CD31+ angiogenesis, and PD-L1 expression versus control; E→S and S→E were also effective. For KiCa-Pt118, S→E and P→E reduced tumor burden (*p* < 0.01) and Ki67^+^ proliferation; S→E lowered CD31 and PD-L1. **Conclusions:** This RCC PDOX platform faithfully preserves patient-specific biology—including metastatic propensity, engraftment efficiency, growth kinetics, and stromal dependency—while enabling real-time evaluation of sequential targeted therapies. Given the limited number of models tested, these findings provide proof-of-concept for individualized treatment exploration in advanced RCC and support future investigation of rational combinations with immune checkpoint blockade in humanized or immunocompetent systems.

## Simple Summary

Advanced kidney cancer (renal cell carcinoma) is difficult to treat because patients respond differently to the same drugs, and the cancer often becomes resistant over time. Doctors currently do not know the best order in which to give available targeted medicines. In this study, we created personalized “avatar” models by implanting tumor samples from two patients with advanced kidney cancer into special mice. These models closely copied each patient’s cancer behavior, including how fast it grew and whether it spread to the lungs. We then tested different sequences of three commonly used targeted drugs in these avatars. The results showed that the best drug sequence differed between the two patients, matching their individual disease courses. This approach may help doctors choose more effective drug sequences for each patient and guide future combinations with immune therapies. Our findings demonstrate that patient-derived avatar models can serve as a useful tool to personalize treatment for advanced kidney cancer.

## 1. Introduction

Renal cell carcinoma (RCC) accounts for approximately 4% of all newly diagnosed cancers in the United States and is associated with poor outcomes in advanced disease [1]. In 2026, an estimated 80,450 new cases of kidney and renal pelvis cancer are projected (50,770 in men and 29,680 in women), with 15,160 deaths (10,200 in men and 4960 in women) [1]. The overall 5-year relative survival rate for RCC is approximately 79% (based on Surveillance, Epidemiology, and End Results Program [SEER] data from 2015–2021), with localized disease carrying an excellent prognosis (~93%) but metastatic RCC (mRCC) having a dismal outlook (~19%) [1]. Over 30% of patients present with de novo metastatic disease, and an additional 30–40% develop local or distant metastases after initial diagnosis [2,3,4].

Although surgery remains the cornerstone for localized disease, systemic therapy is the mainstay for advanced RCC. Over the past two decades, targeted therapies—including multi-tyrosine kinase inhibitors (TKIs) such as sunitinib and pazopanib, and mTOR inhibitors such as everolimus—have improved outcomes [5,6,7]. However, responses vary significantly between patients, and resistance frequently develops. Response rates, median progression-free survival (PFS), and median overall survival (OS) with these first-line targeted therapies are typically modest: 22–29%, 5–9 months, and 12–28 months, respectively [8,9]. While contemporary first-line treatment is now dominated by immunotherapy/TKI combinations and dual immune checkpoint inhibition, optimal sequencing of targeted agents remains clinically relevant, particularly in later lines of therapy or for patients who are ineligible for or progress on immunotherapy [10,11].

In the pre-immune checkpoint inhibitor era, the most commonly employed sequences involved VEGF TKIs (sunitinib or pazopanib) followed by mTOR inhibition (everolimus), supported by pivotal trials such as RECORD-1 (everolimus post-TKI failure) and RECORD-3 (sunitinib→everolimus vs. everolimus→sunitinib) [12,13]. TKI-to-TKI switches (e.g., pazopanib→sunitinib) were also explored in real-world settings due to differential tolerability or resistance profiles [14]. To model these clinically relevant patterns and test bidirectional sequencing in a patient-specific manner, we evaluated four combinations—Everolimus→Sunitinib (E→S), Pazopanib→Sunitinib (P→S), Sunitinib→Everolimus (S→E), and Pazopanib→Everolimus (P→E)—in our patient-derived orthotopic xenograft (PDOX) avatar platform.

Conventional RCC cell lines fail to capture tumor heterogeneity and tumor–microenvironment interactions. Patient-derived xenografts (PDX) better preserve tumor architecture and genomic integrity. Orthotopic PDX models, PDOXs, in particular, more accurately recapitulate metastatic progression [15,16]. While 3D scaffold culturing enables sophisticated cell–cell interactions (including with immune and neuronal cells), it often requires specialized bioengineering expertise and access to advanced laboratory infrastructure [17,18]. PDX models represent the most reliable and well-studied approach to personalized medicine for predicting clinical responses to therapy. PDX tumors retain parental tumor histology, architecture, and genomic composition [19,20,21]. Tumorigenesis occurs in an environment with a functional endocrine system and a partially functional mouse immune system [20]. Disease progression in the mouse closely resembles that of the patient [20]. PDOXs are particularly efficient in mimicking patient disease progression in RCC [22].

Here, we developed a luciferase-enabled RCC PDOX platform that permits real-time monitoring of tumor growth and metastasis. This platform was used to evaluate sequential targeted therapy strategies in individualized mouse avatars derived from two clinically divergent RCC patients.

## 2. Materials and Methods

### 2.1. Patient Specimens

Tumor samples from two consenting patients with RCC were obtained immediately following radical nephrectomy performed by the Urology Department at Ochsner Medical Center. The study was approved by the Institutional Review Board of Ochsner Medical Center (protocol details available upon request). Histological diagnoses were confirmed by a board-certified pathologist (Table 1). Clinical outcomes further distinguished the cases: Patient KiCa-Pt58 (kidney cancer patient 58, metastatic sarcomatoid RCC, pT3aN1M1) succumbed to disease one month post-nephrectomy, whereas Patient KiCa-Pt118 (kidney cancer patient 118, clear cell RCC with sarcomatoid component, pT3aNxM0) remained recurrence-free with no evidence of disease >8 years post-nephrectomy, and passed away from a heart attack.

### 2.2. Establishment of Luciferase-Tagged Xenografts

Fresh tumor tissues were mechanically minced into 2–3 mm^3^ fragments and implanted subcutaneously into NOD/SCID mice. Once tumors reached approximately 1 cm in diameter, they were transduced intratumorally with a lentiviral construct encoding luciferase and TurboRFP under CMV promoters (tNL(CMV)Luc2-TurboRFP/CMV/WPREΔU3) [23,24]. Luc/RFP expression was monitored weekly by BLI. Tumors with strong signal were serially passaged by implanting the highest-expressing fragments into additional mice. Tumors exhibiting >80% RFP positivity (determined by fluorescence-activated cell sorting [FACS]) were enzymatically dissociated into single-cell suspensions for subsequent orthotopic PDOX establishment [24,25].

### 2.3. Orthotopic PDOX Model

All animal procedures were approved by the Institutional Animal Care and Use Committee (IACUC) of Ochsner Clinic Foundation and conducted in accordance with NIH guidelines for animal research. NOD/SCID mice were bred in-house from Jackson Laboratory stock (Bar Harbor, ME, USA) and used at 10–14 weeks of age. Both male and female mice were used. After confirmation of tumor establishment by bioluminescence imaging (BLI), animals were randomized into treatment groups to achieve comparable baseline tumor burden between groups. Investigators performing BLI quantification and histological/immunohistochemical analyses were not formally blinded to treatment allocation. No formal a priori sample-size power calculation was performed; group sizes (*n* = 7–9 per group) were selected based on prior experience with PDOX therapeutic studies and feasibility considerations. Animals were monitored at least three times weekly for body weight, general condition, activity, grooming behavior, and signs of treatment-related toxicity. Humane endpoint criteria included excessive tumor burden, marked weight loss (>15–20%), impaired mobility, lethargy, or ulceration, in accordance with IACUC guidelines. Animals with unsuccessful tumor engraftment or inadequate baseline BLI signal prior to treatment initiation were excluded from therapeutic analysis.

Tumor fragments were enzymatically digested using DNase I (0.1 mg/mL), collagenase IV (1.5 mg/mL), and hyaluronidase (20 mg/mL; all Sigma-Aldrich, St. Louis, MO, USA) in Hank’s Balanced Salt Solution (HBSS) with gentle shaking at 37 °C for 2–3 h. Digests were filtered through a 40 μm cell strainer (Corning, Corning, NY, USA) to obtain single-cell suspensions.

Luc/RFP-tagged cells were injected subcapsularly into the left kidney of anesthetized mice (isoflurane 3% in 100% O_2_ at 1 L/min). Briefly, hair on the left flank was removed with Nair™, and a sterile flank incision exposed the kidney. For KiCa-Pt58: 4 × 10^4^, 2 × 10^5^, and 1 × 10^6^ cells, all in 10 µL RPMI, were injected; for KiCa-Pt118: 3 × 10^5^ and 1 × 10^6^ cells were co-injected with human lymph node stromal cells (HK, 3 × 10^5^ cell per mouse, all in 10 µL RPMI) to support engraftment. The kidney was returned to the abdominal cavity, and the muscle and skin layers were sutured close. Mice received buprenorphine SR (0.5 mg/kg) postoperatively and were monitored daily for 3 days, then on days 7 and 14.

Tumor progression and metastasis were monitored weekly using the IVIS Lumina Imaging System (PerkinElmer, Waltham, MA, USA) and Living Image software v.4.5 (Caliper Life Sciences, Hopkinton, MA, USA). Luciferin (15 mg/mL) was administered intraperitoneally (IP, 150 mg/kg), followed by a 5 min uptake period in awake mice. Mice were then anesthetized (3% isoflurane), imaged at auto-exposure, and total flux (photons/s) was quantified from regions of interest (ROI). Animals were euthanized at IACUC-defined humane endpoints or when primary tumor BLI reached predetermined thresholds. At necropsy, lungs, liver, and both kidneys were excised, weighed, imaged ex vivo by the IVIS system, fixed in formalin, and embedded in paraffin for hematoxylin and eosin (H&E) and immunohistochemistry (IHC) analysis.

### 2.4. Sequential Drug Treatment

PDOX models were established using KiCa-Pt58 (1 × 10^4^ cell per mouse, *n* = 42 mice) and KiCa-Pt118 (3 × 10^5^ cells plus 3 × 10^5^ HK cells per mouse, *n* = 39 mice). After initial BLI confirmation of tumor establishment, mice were randomized into five groups: vehicle control (water) or one of four sequential treatment arms (Everolimus→Sunitinib [E→S], Sunitinib→Everolimus [S→E], Pazopanib→Sunitinib [P→S], Pazopanib→Everolimus [P→E]; *n* = 7–9 per group). Treatment doses were Everolimus 5 mg/kg, Sunitinib 40 mg/kg, or Pazopanib 40 mg/kg, administered by oral gavage three times weekly. These doses were previously determined to be safe and effective without overt toxicity. Treatment started on day 7 post-injection for KiCa-Pt58 models and day 50 for KiCa-Pt118 models. Tumor response was monitored weekly by BLI. Upon a >200% increase in BLI signal from previous week (a sign of drug resistance), treatment was switched once to the second agent. This operational threshold was selected as a pragmatic indicator of progressive tumor burden in the context of longitudinal preclinical BLI monitoring, where fold-changes in photon flux are commonly used to track tumor progression and therapeutic response in real time [26,27]. While week-to-week variability in BLI measurements may influence a moving-reference approach, this strategy enabled timely adaptation to emerging resistance in the avatar setting. Future studies may incorporate nadir-based or smoothed progression criteria to improve robustness and reproducibility. Switches occurred between days 14–28 for KiCa-Pt58 and days 57–64 for KiCa-Pt118. Mice were euthanized on day 36 (KiCa-Pt58) or day 77 (KiCa-Pt118) post-injection. Primary tumor burden (BLI and weight at necropsy), lung metastatic burden (ex vivo BLI), and histological/IHC at the endpoints were recorded and analyzed to evaluate sequential regimen efficacy.

### 2.5. Histological and Immunohistochemical Analysis

Paraffin-embedded sections (5 μm) were stained with H&E or subjected to immunohistochemistry using primary antibodies against human Ki67 (proliferation marker; Thermo Fisher Scientific, Waltham, MA, USA; 1:200), human CD44 (tumor cell marker; Acris Antibodies, Rockville, MD, USA; 1:75), mouse CD31 (angiogenesis marker; Abcam, Cambridge, MA, USA; 1:200), and human PD-L1 (programmed death-ligand 1, immune checkpoint ligand; BioLegend, San Diego, CA, USA; 1:200) [25]. Images were captured at 100× magnification using an Axiovert 200M deconvolution microscope and SlideBook 6.0 software (Intelligent Imaging Innovations, Denver, CO, USA). Positive (brown) staining areas were quantified digitally using Adobe Photoshop 7.0 by calculating the percentage of immunoreactive areas per field. Data were analyzed statistically as described below.

### 2.6. Statistical Analysis

Data are presented as mean ± standard error of the mean (SEM). Statistical significance was determined using unpaired two-tailed *t*-tests for two-group comparisons or one-way ANOVA with appropriate post hoc tests (e.g., Tukey’s or Dunnett’s) for multiple groups (GraphPad Prism version 7; GraphPad Software, La Jolla, CA, USA). Chi-squared (χ^2^) tests were used for categorical data where applicable. A *p*-value < 0.05 was considered statistically significant (* *p* < 0.05; ** *p* < 0.01; *** *p* < 0.001). All experiments were performed with biological replicates and independently reproduced at least once.

## 3. Results

### 3.1. Successful Establishment of RCC PDOX Models

**Figure 1:**
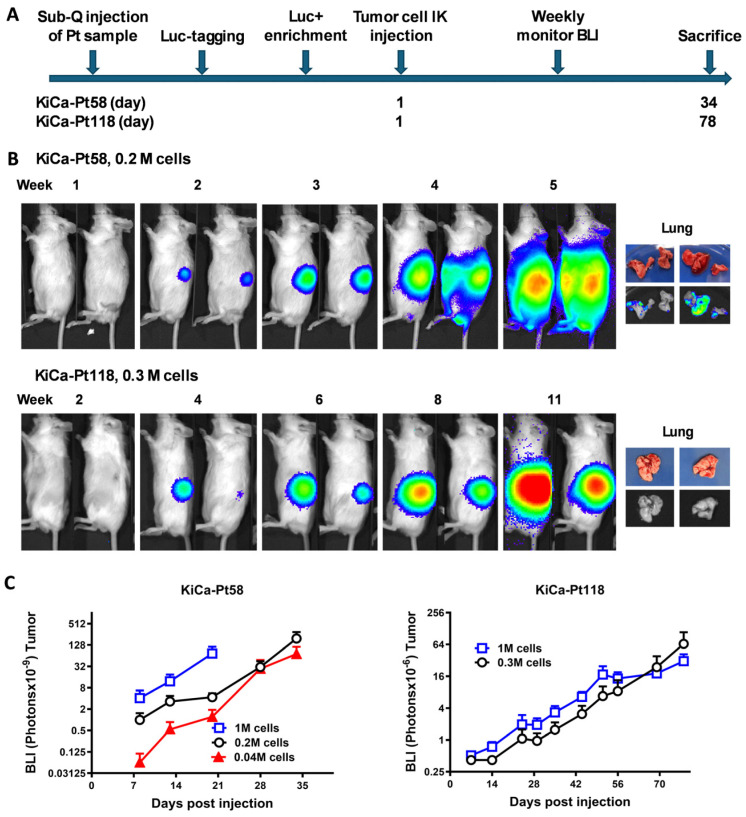
Establishment and engraftment optimization of patient-derived orthotopic xenograft (PDOX) models for renal cell carcinoma (RCC). (**A**) Schematic timeline of the workflow for PDOX model development and tumor cell dosing optimization. Fresh patient tumor samples were implanted subcutaneously in NOD/SCID mice for expansion, followed by lentiviral luciferase/red fluorescent protein (Luc/RFP) transduction, enrichment of Luc^+^ cells, orthotopic (subcapsular) injection into the left kidney (intra-kidney sub-capsular [IK] injection) at varying doses, weekly bioluminescence imaging (BLI) monitoring, and euthanasia at endpoint. (**B**) Representative in vivo BLI images of primary tumor growth in the left kidney over selected weeks, plus ex vivo lung images at necropsy (macroscopic photos and BLI signal). For KiCa-Pt58 (sarcomatoid RCC, metastatic; aggressive biology), representative images after injection of 0.2 × 10^6^ tumor cells (no stromal support) show rapid engraftment (detectable by week 2), progressive primary tumor growth (high-intensity signal by week 5), and spontaneous lung metastases (visible macroscopic nodules and positive ex vivo BLI in lungs). For KiCa-Pt118 (clear cell RCC with sarcomatoid component, non-metastatic; indolent biology), representative images after injection of 0.3 × 10^6^ tumor cells (with human lymph node stromal [HK] support) show delayed engraftment (detectable by week 4), slower primary growth to week 11, and no detectable lung metastases ex vivo. Quantitative primary tumor burden is reported as total flux (photons/s) over time. (**C**) Quantitative BLI signals (total flux in photons/s) for primary tumor burden over the full experimental time course post-injection, summarizing all tested cell doses (mean ± SEM; *n* = 5–6 mice per condition). KiCa-Pt58: fast, dose-dependent engraftment (0.04 × 10^6^, 0.2 × 10^6^, 1 × 10^6^ cells; all with no HK cells), with robust signal increase, consistent with the patient’s metastatic sarcomatoid disease and rapid clinical demise. KiCa-Pt118: slower kinetics at 0.3 × 10^6^ cells (with HK stromal cell co-injection), mirroring the patient’s prolonged recurrence-free survival (>8 years post-nephrectomy). Abbreviations: PDOX, patient-derived orthotopic xenograft; RCC, renal cell carcinoma; BLI, bioluminescence imaging; Luc^+^, luciferase-positive; IK, intra-kidney sub-capsular; HK, human lymph node stromal cell.

To develop a preclinical model for screening targeted therapies in RCC, we established PDOX models using specimens from two patients (Table 1). Tumor fragments were subcutaneously engrafted in NOD/SCID mice, transduced with luciferase/red fluorescent protein (Luc/RFP), enriched for Luc^+^/RFP^+^ cells, and injected subcapsularly into the left kidney as single-cell suspensions (Figure 1A). Tumor engraftment and growth were monitored weekly by BLI.

To assess potential effects of Luc/RFP lentiviral transduction on tumor growth, we compared tagged and non-tagged tumors in a closely related RCC model (KiCa-Pt431, clear cell RCC) using subcutaneous injection. KiCa-Pt431 was selected for this validation because it reproducibly engrafts subcutaneously in our PDOX platform. As shown in Supplementary Figure S1, both lines successfully formed tumors. Although measurements were taken on slightly different schedules, the non-tagged tumors tended to exhibit faster progression in the later stages. Overall, both tagged and non-tagged tumor growth in this experiment fell within the expected range for the KiCa RCC PDOX models evaluated in this manuscript (e.g., KiCa-Pt58 and KiCa-Pt118) under comparable implantation conditions. Histological comparison showed no major differences. These findings indicate that Luc/RFP tagging imposes only a modest growth disadvantage and does not fundamentally compromise the utility of the PDOX platform.

Detectable tumors appeared in most animals by week 2 for KiCa-Pt58 and week 4 for KiCa-Pt118 (Figure 1B). Engraftment rates were 87% for KiCa-Pt58 and 77% for KiCa-Pt118. Mice were euthanized when IACUC-defined endpoints were reached due to tumor burden (day 34 for KiCa-Pt58; day 78 for KiCa-Pt118). Ex vivo BLI of lungs at necropsy revealed spontaneous lung metastases in 78% of KiCa-Pt58-bearing mice, but none in KiCa-Pt118-bearing mice—mirroring the patients’ clinical profiles (metastatic sarcomatoid RCC in KiCa-Pt58 vs. non-metastatic clear cell RCC with sarcomatoid features in KiCa-Pt118; Table 1).

The aggressive, metastatic phenotype of KiCa-Pt58 was faithfully recapitulated: high engraftment efficiency at low cell doses (4 × 10^4^ cells, no stromal support, Figure 1C), rapid progression (detectable by week 2), and frequent lung metastases. Although the patient primarily exhibited adrenal metastasis (pT3aN1M1), the PDOX model similarly demonstrated preservation of the highly aggressive metastatic behavior of the parental sarcomatoid RCC. In contrast, KiCa-Pt118 PDOX tumors required higher cell numbers (3 × 10^5^ cells) with human lymph node stromal (HK) cell co-injection for reliable engraftment (Figure 1C) [24,25,28,29], exhibited delayed growth, and showed no metastases—paralleling the patient’s prolonged (>8 year) recurrence-free survival.

### 3.2. PDOX Tumors Recapitulate Parental Histology

**Figure 2:**
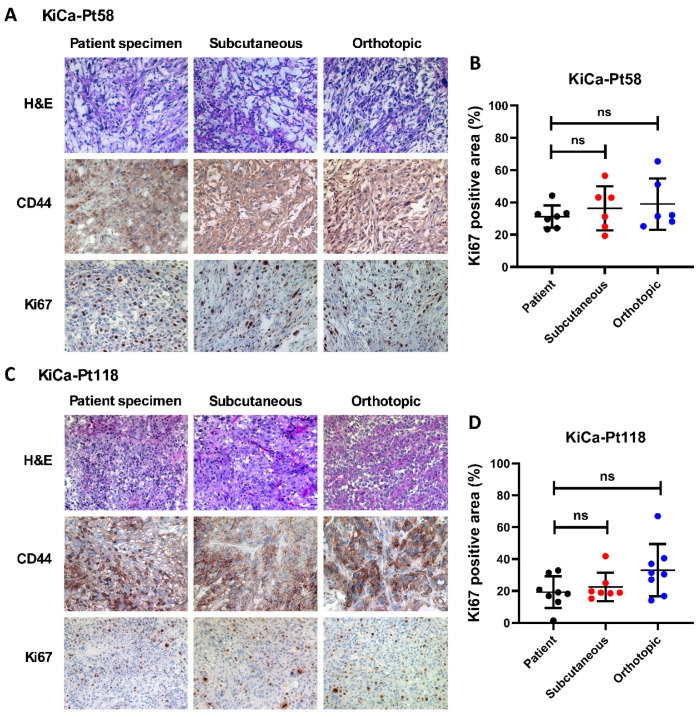
Histopathological comparison of tumor architecture and proliferation between patient renal cell carcinoma (RCC) specimens and corresponding patient-derived xenograft models. Formalin-fixed, paraffin-embedded sections from primary patient tumors (KiCa-Pt58 and KiCa-Pt118), matched subcutaneous xenografts, and intra-renal patient-derived orthotopic xenograft (PDOX) tumors were analyzed to assess preservation of tumor histology and proliferative characteristics. (**A**,**C**) Representative images of KiCa-Pt58 (**A**) and KiCa-Pt118 (**C**) tissues stained with hematoxylin and eosin (H&E) for tissue architecture or were subjected to immunohistochemistry (IHC) for human CD44 (tumor cell marker) and human Ki67 (proliferation marker). Brown staining indicates positive immunoreactivity. Images were captured at 100× original magnification using an Axiovert 200M deconvolution microscope and SlideBook 6.0 software (Intelligent Imaging Innovations, Denver, CO, USA). (**B**,**D**) Quantitative analysis of Ki67-positive area (%) in KiCa-Pt58 (**B**) and KiCa-Pt118 (**D**) tissues. Positive (brown) staining areas were quantified digitally using Adobe Photoshop 7.0, with the percentage of immunoreactive area calculated per field. Data are presented as mean ± SEM (multiple fields per sample). Comparisons among patient biopsy specimens, subcutaneous xenografts, and orthotopic PDOX tumors were performed using unpaired Student’s *t*-test. No significant differences were observed (ns, *p* > 0.05), demonstrating faithful recapitulation of the parental tumor proliferative index across model passages. Abbreviations: RCC, renal cell carcinoma; PDOX, patient-derived orthotopic xenograft; H&E, hematoxylin and eosin; IHC, immunohistochemistry.

To confirm fidelity to the original tumors, we performed histological and immunohistochemical analyses on patient biopsies, early-passage subcutaneous xenografts (passage 2–4), and orthotopic PDOX tumors (passage 1 after subcutaneous expansion). H&E staining demonstrated consistent tumor architecture across passages for both KiCa-Pt58 and KiCa-Pt118 (Figure 2A,C). Immunohistochemistry for human CD44 (tumor cell marker) and Ki67 (proliferation marker) revealed preserved expression patterns, with highly proliferative human tumor cells evident in the mouse models. Quantitative analysis Ki67^+^ area (mean of 6–12 random high-power fields per sample) showed no significant differences between patient specimens, subcutaneous xenografts, and PDOX tumors for either case (Figure 2B,D; *p* > 0.05, unpaired two-tailed *t*-test). While representative images suggest minor variability in staining intensity (likely due to regional tumor heterogeneity or tissue processing), these results confirm that our PDOX platform maintains key histological features, proliferative index, and CD44 expression from the parental tumors through serial passaging. Importantly, Luc/RFP labeling was not associated with obvious changes in baseline tumor features: histological architecture and Ki67 proliferative index were preserved across patient biopsy, subcutaneous xenograft, and orthotopic PDOX tissues (Figure 2; *p* > 0.05). Each model also retained its expected case-specific in vivo behavior, including differential engraftment requirements, growth rate, and metastatic patterns consistent with the corresponding patient clinical course.

### 3.3. Differential Responses to Sequential Targeted Therapy

**Figure 3:**
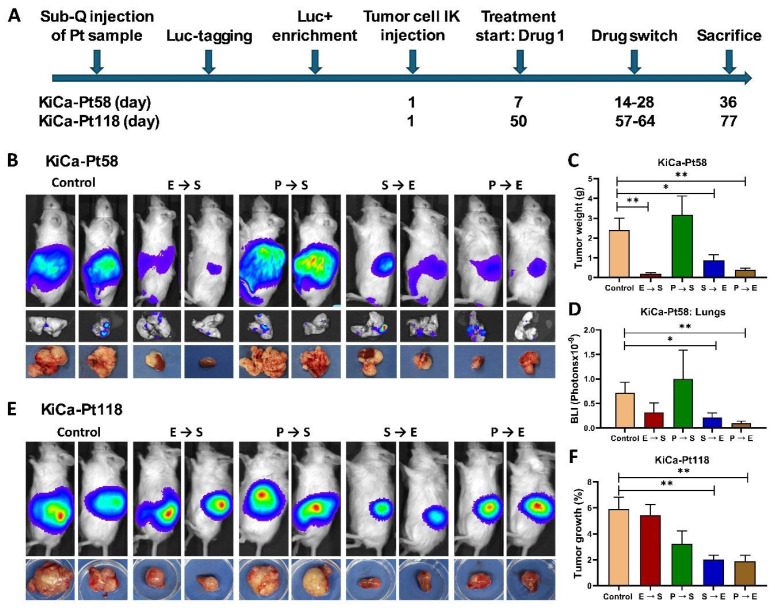
Therapeutic response profiling of renal cell carcinoma (RCC) patient-derived orthotopic xenograft (PDOX) mouse avatars to sequential targeted therapies. (**A**) Schematic timeline of the PDOX workflow and sequential treatment protocol. After model establishment (subcutaneous expansion, Luc/RFP tagging, enrichment, and orthotopic intra-kidney sub-capsular [IK] injection), tumors were monitored by bioluminescence imaging (BLI). Treatment began with Drug 1 upon detectable tumor signal, with a single switch to a second agent upon resistance (>200% BLI increase), followed by euthanasia at endpoint. (**B**,**E**) Representative in vivo BLI images of primary tumor burden and corresponding ex vivo tumor photographs at necropsy for KiCa-Pt58 (**B**) and KiCa-Pt118 (**E**). Mice were randomized to vehicle control or sequential regimens: Everolimus→Sunitinib (E→S), Pazopanib→Sunitinib (P→S), Sunitinib→Everolimus (S→E), or Pazopanib→Everolimus (P→E). (**C**,**D**) For KiCa-Pt58 (aggressive, metastatic model): primary tumor burden quantified by tumor weight at sacrifice (**C**) and lung metastatic burden by ex vivo BLI (**D**). The P→E sequence showed the greatest reduction in tumor weight (** *p* < 0.01) and lung metastases (** *p* < 0.01) compared to control; E→S and S→E also conferred significant benefits. (**F**) For KiCa-Pt118 (indolent, non-metastatic model): tumor response expressed as percentage growth inhibition relative to control. S→E and P→E sequences significantly inhibited tumor growth (** *p* < 0.01). Data are presented as mean ± SEM (*n* = 7–9 mice per group). Statistical significance was determined using one-way ANOVA with appropriate post hoc multiple comparison tests (GraphPad Prism v7). Asterisks indicate significance vs. control: * *p* < 0.05; ** *p* < 0.01. Abbreviations: RCC, renal cell carcinoma; PDOX, patient-derived orthotopic xenograft; BLI, bioluminescence imaging; E, everolimus; S, sunitinib; P, pazopanib.

We evaluated sequential targeted therapy responses in the PDOX avatars using four regimens combining sunitinib (S), pazopanib (P), and everolimus (E): E→S, P→S, S→E, and P→E, with vehicle control. Mice were treated orally three times weekly starting upon detectable tumor signal, with a single drug switch upon resistance (>200% BLI increase). Tumor burden was monitored weekly by BLI (Figure 3A).

For KiCa-Pt58 (aggressive, metastatic model), three of four sequences significantly reduced primary tumor weight at sacrifice (E→S: *p* = 0.0018; S→E: *p* = 0.0337; P→E: *p* = 0.0059; Figure 3B,C). P→E produced the greatest overall reductions in both primary tumor weight and lung metastases (** *p* < 0.01 vs. control for both), followed by E→S (strongest on tumor weight) and S→E. Lung metastatic burden (ex vivo BLI) was also significantly decreased by S→E (*p* = 0.0394) and P→E (*p* = 0.008; Figure 3D), with P→E showing the strongest effect.

For KiCa-Pt118 (indolent, non-metastatic model), S→E and P→E significantly inhibited tumor growth relative to control (*p* = 0.002 and *p* = 0.0038, respectively; Figure 3E,F). No metastases were observed in this model, consistent with the patient’s clinical course.

Overall, P→E was most effective against primary tumor progression and lung metastases in KiCa-Pt58, while S→E and P→E were most effective in KiCa-Pt118—highlighting patient-specific differential responses. These PDOX behaviors (metastatic propensity, engraftment efficiency, stromal dependency, growth kinetics) directly mirrored the patients’ outcomes (rapid demise of KiCa-Pt58 vs. long-term remission of KiCa-Pt118), reinforcing the model’s utility as a personalized avatar for guiding sequential therapy.

**Figure 4:**
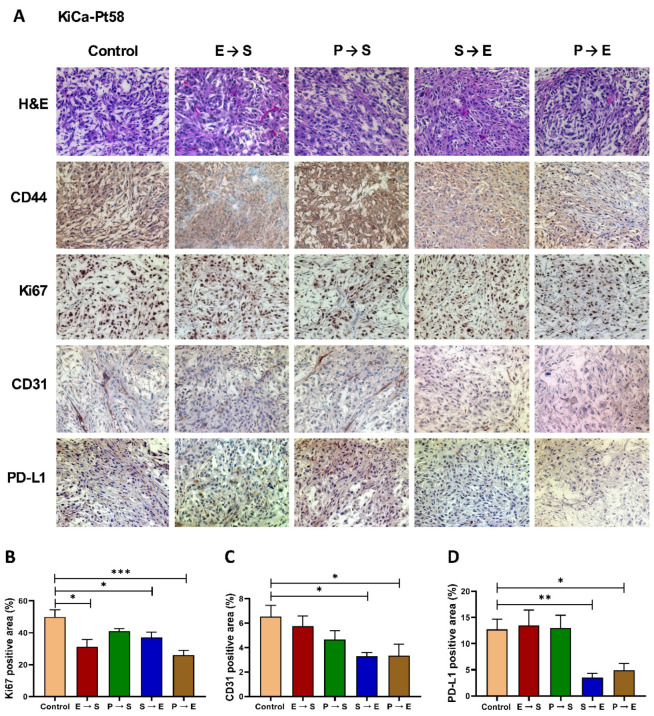
Histopathological and immunohistochemical evaluation of targeted therapy responses in the KiCa-Pt58 patient-derived orthotopic xenograft (PDOX) model. (**A**) Representative images of left kidney tumors from KiCa-Pt58 PDOX mice after vehicle control or sequential targeted therapy (Everolimus→Sunitinib [E→S], Pazopanib→Sunitinib [P→S], Sunitinib→Everolimus [S→E], Pazopanib→Everolimus [P→E]). Formalin-fixed, paraffin-embedded sections were stained with hematoxylin and eosin (H&E) for tumor architecture or subjected to immunohistochemistry (IHC) for human CD44 (tumor cell marker), human Ki67 (proliferation marker), mouse CD31 (angiogenesis/endothelial marker), and human PD-L1 (immune checkpoint ligand). Brown staining indicates positive immunoreactivity. Images were acquired at 100× original magnification using an Axiovert 200M deconvolution microscope and SlideBook 6.0 software (Intelligent Imaging Innovations, Denver, CO, USA). (**B**–**D**) Quantitative analysis of positive staining area (%) for Ki67 (**B**), CD31 (**C**), and PD-L1 (**D**) across treatment groups. Positive (brown) areas were quantified digitally using Adobe Photoshop 7.0 (percentage immunoreactive area per field). Data are presented as mean ± SEM (multiple fields per sample; *n* = 7–9 mice per group). Statistical comparisons vs. control were performed using one-way ANOVA followed by Dunnett’s or Tukey’s post hoc tests (GraphPad Prism v7). Asterisks indicate significance: * *p* < 0.05; ** *p* < 0.01; *** *p* < 0.001. Effective regimens (particularly P→E and S→E) significantly reduced Ki67+ proliferation, CD31+ vascularity, and PD-L1 expression compared to control, consistent with antitumor and potential immunomodulatory effects. Abbreviations: PDOX, patient-derived orthotopic xenograft; IHC, immunohistochemistry; E, everolimus; S, sunitinib; P, pazopanib.

**Figure 5:**
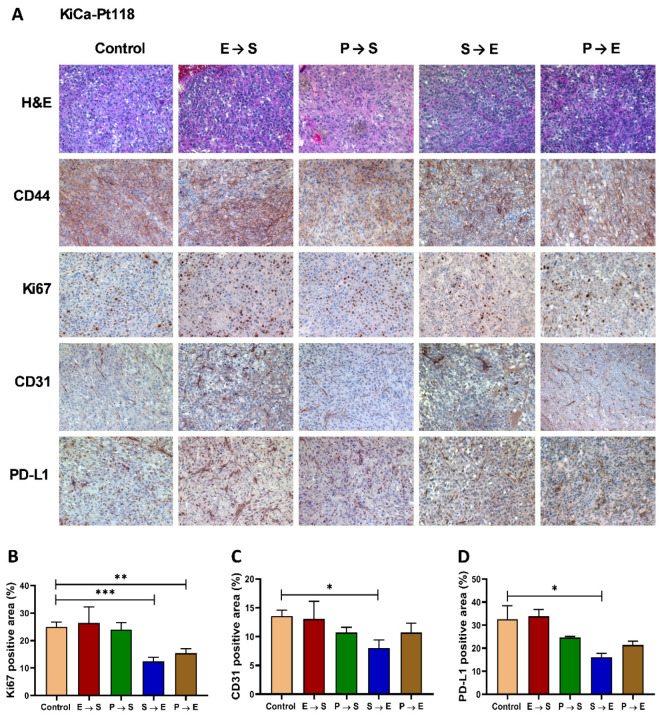
Histopathological and immunohistochemical assessment of targeted therapy responses in the KiCa-Pt118 patient-derived orthotopic xenograft (PDOX) model. (**A**) Representative images of left kidney tumors from KiCa-Pt118 PDOX mice after vehicle control or sequential targeted therapy (Everolimus→Sunitinib [E→S], Pazopanib→Sunitinib [P→S], Sunitinib→Everolimus [S→E], Pazopanib→Everolimus [P→E]). Formalin-fixed, paraffin-embedded sections were stained with hematoxylin and eosin (H&E) for tumor architecture or subjected to immunohistochemistry (IHC) for human CD44 (tumor cell marker), human Ki67 (proliferation marker), mouse CD31 (angiogenesis/endothelial marker), and human PD-L1 (immune checkpoint ligand). Brown staining indicates positive immunoreactivity. Images were acquired at 100× original magnification using an Axiovert 200M deconvolution microscope and SlideBook 6.0 software (Intelligent Imaging Innovations, Denver, CO, USA). (**B**–**D**) Quantitative analysis of positive staining area (%) for Ki67 (**B**), CD31 (**C**), and PD-L1 (**D**) across treatment groups. Positive (brown) areas were quantified digitally using Adobe Photoshop 7.0 (percentage immunoreactive area per field), as described in Figure 4. Data are presented as mean ± SEM (multiple fields per sample; *n* = 7–9 mice per group). Statistical comparisons vs. control were performed using one-way ANOVA followed by Dunnett’s or Tukey’s post hoc tests (GraphPad Prism v7). Asterisks indicate significance: * *p* < 0.05; ** *p* < 0.01; *** *p* < 0.001. Effective regimens (particularly S→E) significantly reduced Ki67+ proliferation, CD31+ vascularity, and PD-L1 expression compared to control, consistent with antitumor activity in this indolent, non-metastatic model. Abbreviations: PDOX, patient-derived orthotopic xenograft; IHC, immunohistochemistry; E, everolimus; S, sunitinib; P, pazopanib.

Histological and IHC analyses further validated these responses. H&E staining revealed improved tumor architecture and nuclear regularity in KiCa-Pt58 tumors treated with E→S and S→E compared to control (Figure 4A). In KiCa-Pt118, S→E treatment notably improved architecture and nuclear morphology (Figure 5A, H&E, and CD44). Ki67 IHC showed significant reductions in proliferation in effective treatment groups: for KiCa-Pt58, E→S (*p* = 0.0141), S→E (*p* = 0.0384), and especially P→E (*p* = 0.0004; Figure 4B); for KiCa-Pt118, S→E (*p* = 0.0006) and P→E (*p* = 0.0059; Figure 5B). CD31 staining (angiogenesis) was significantly reduced by S→E (*p* = 0.0172) and P→E (*p* = 0.0306) in KiCa-Pt58 (Figure 4C), and by S→E (*p* = 0.0113) in KiCa-Pt118 (Figure 5C).

### 3.4. PD-L1 Expression and Implications for Immunotherapy

PD-L1 expression was assessed by IHC. Baseline PD-L1 positivity was 12.7% in KiCa-Pt58 (Figure 4D) and 32.6% in KiCa-Pt118 (Figure 5D). Effective sequences significantly reduced PD-L1^+^ area: in KiCa-Pt58, S→E (to 3.5%; *p* = 0.003) and P→E (to 4.9%; *p* = 0.0116); in KiCa-Pt118, S→E (to 16.2%; *p* = 0.0454). These reductions suggest that targeted therapy may modulate the immune microenvironment, potentially enhancing sensitivity to immune checkpoint blockade (ICB). Both models—and by extension both patients—may benefit from TKI/mTOR inhibitor plus ICB combinations, which are increasingly used as first-line therapy in advanced RCC.

The combination of BLI, tumor burden, and IHC data (summarized in Table 2) demonstrates that our PDOX platform can identify patient-specific therapeutic vulnerabilities, supporting individualized strategies in RCC.

### 3.5. Clinical Correlation

KiCa-Pt58

This 56-year-old male presented with advanced sarcomatoid RCC with focal chromophobe differentiation (pT3aN1M1, Fuhrman grade 4, 17.5 × 15 cm tumor). The aggressive sarcomatoid histology and metastatic presentation at diagnosis conferred a poor prognosis (historical median OS 6–13 months post-nephrectomy in metastatic sRCC). The patient succumbed to the disease one month post-surgery. The PDOX model faithfully recapitulated this fulminant course: high engraftment at low cell doses (1 × 10^4^ cells, no stromal support), rapid tumor detection (week 2), accelerated progression, and frequent spontaneous lung metastases (78% of mice), leading to early euthanasia (day 36).

KiCa-Pt118

This 71-year-old male had clear cell RCC with sarcomatoid component (pT3aNxM0, Fuhrman grade 4, 8.0 × 6.5 cm tumor). Despite sarcomatoid dedifferentiation (an adverse feature increasing recurrence risk), the patient remained recurrence-free for >8 years post-surgery and ultimately died of a non-cancer cause (heart attack), indicating indolent biology in this non-metastatic case. The PDOX model mirrored this profile: no lung metastases, delayed engraftment requiring higher cell inoculum (3 × 10^5^ cells) plus human lymph node stromal (HK) cell support, slower growth (detection by week 4), and later euthanasia (day 77). These differences underscore the PDOX platform’s ability to capture patient-specific metastatic potential, stromal dependency, and disease kinetics.

## 4. Discussion

Mouse models remain essential for elucidating the biology and molecular mechanisms of RCC tumorigenesis and progression. Although PDX models have been widely used in basic research, their clinical translation has been limited by several challenges. First, PDX establishment is time-consuming, often requiring multiple serial passages to generate sufficient tumor material. Second, engraftment rates vary widely (20–80%), depending on tumor stage and subtype [30]. Orthotopic implantation (PDOX models) partially overcomes these limitations by better recapitulating the native tumor microenvironment and metastatic patterns in RCC [31]. Orthotopic RCC PDOX models have proven superior to subcutaneous PDX in mimicking patient disease progression and distant metastasis [22].

A third major barrier has been real-time, high-throughput visualization of tumor burden in orthotopic models. Conventional imaging modalities (ultrasound, CT, PET/CT, MRI) are low-throughput and unsuitable for serial monitoring of multiple animals [32,33]. Bioluminescence imaging offers excellent sensitivity and throughput but has been rarely applied in RCC PDOX due to difficulties in stable luciferase labeling of primary patient-derived cells. Prior approaches typically require establishing patient-derived cell lines before lentiviral transduction [34].

In this study, we developed a novel RCC PDOX platform that circumvents these hurdles by direct lentiviral Luc/RFP transduction of subcutaneous xenografts, followed by selection of high-expressing populations and orthotopic implantation of single-cell suspensions [24,25]. This method is straightforward, does not require long-term in vitro culture or specialized bioengineering, and uses commercially available lentivirus. High Luc/RFP expression enabled accurate, weekly BLI monitoring of tumor growth and spontaneous metastasis in individual patient avatars (KiCa-Pt58 and KiCa-Pt118) using the IVIS Lumina system.

To evaluate potential effects of transduction, we compared Luc/RFP-tagged and non-tagged tumors in a closely related RCC model (KiCa-pt431). Tagged tumors showed comparable early growth but moderately slower progression in later stages compared to non-tagged parental tumors (Supplementary Figure S1). Histological features remained similar. These data suggest that Luc/RFP tagging imposes only a modest growth disadvantage without compromising the overall utility of the PDOX platform.

Our PDOX models faithfully recapitulated patient-specific biology: KiCa-Pt58 (metastatic sarcomatoid RCC) showed rapid engraftment, high metastatic propensity (78% lung metastases), and aggressive progression, while KiCa-Pt118 (non-metastatic clear cell RCC with sarcomatoid features) exhibited delayed growth, stromal dependency, and no metastases—mirroring each of the patients’ clinical courses (rapid demise vs. >8-year recurrence-free survival). Histology, architecture, proliferation (Ki67), and CD44 expression were preserved across passages, underscoring the model’s fidelity. Limiting serial passaging minimized murine stromal replacement and genomic drift, known risks that can alter drug sensitivity [35,36,37].

We applied this platform to evaluate sequential targeted therapies (sunitinib [S], pazopanib [P], everolimus [E]) in four clinically relevant combinations. Optimal sequencing differed markedly between models, reflecting interpatient heterogeneity: P→E was most effective in reducing primary tumor burden and lung metastases in KiCa-Pt58, while S→E and P→E best inhibited growth in KiCa-Pt118. Notably, the P→S sequence in KiCa-Pt58 resulted in accelerated tumor growth compared with untreated controls, suggesting potential cross-resistance or adaptive resistance mechanisms between sequential VEGF-targeted TKIs in this aggressive sarcomatoid RCC model. In contrast, sequences incorporating everolimus after VEGFR inhibition may have partially overcome adaptive signaling escape through mTOR pathway suppression, potentially contributing to the superior efficacy observed with P→E. These responses correlated with reduced proliferation (Ki67), angiogenesis (CD31), and PD-L1 expression in effective regimens. The overall sequence performance by PDOX phenotype is summarized in Table 3.

PD-L1 modulation is particularly relevant in RCC, where ICB agents targeting PD-1 (programmed death receptor-1)/PD-L1 has transformed treatment. The PD-1 receptor on activated T cells interacts with PD-L1 on tumor and antigen-presenting cells to mediate peripheral tolerance and suppress anti-tumor immunity [38,39]. PD-L1 overexpression promotes immune evasion and predicts better ICB responses in RCC [40]. Our findings that effective targeted therapies significantly downregulated PD-L1 suggest potential synergy with ICB, supporting ongoing clinical exploration of TKI/mTOR inhibitor plus ICB combinations as first-line therapy in advanced RCC. Both patient-derived models—and likely the patients—may be strong candidates for such approaches.

While current RCC management relies on surgery followed by sequential targeted agents (often associated with cumulative toxicity), optimal sequencing remains empirical due to tumor heterogeneity. No prior PDOX studies have tested sequential regimens in RCC in vivo. Our platform addresses this gap by providing real-time, patient-specific readouts of therapeutic efficacy and resistance, positioning PDOX avatars as a preclinical decision-support tool for personalized sequencing.

Compared with subcutaneous PDX models, orthotopic implantation better preserved metastatic behavior and tumor architecture. Direct lentiviral labeling of xenografts enables real-time BLI monitoring of tumor growth and metastasis without the need for long-term in vitro cell line establishment.

Current management of RCC includes surgery to remove resectable disease, especially when the cancer is localized, and sequential systemic therapies for inoperable or advanced diseases. Over the last several years, combinations of immunotherapies and VEGF-targeted therapies, along with an immunotherapy doublet (nivolumab plus ipilimumab), have become standard first-line regimens. These combinations have demonstrated superior progression-free survival and overall survival compared with single-agent targeted therapies such as sunitinib in large, randomized phase 3 trials [40,41,42,43]. While sunitinib use has declined in favor of newer TKIs, selecting the most optimal therapy combination in the first-line setting remains a major clinical challenge. Likewise, optimal sequencing of subsequent lines of therapy remains undefined, underscoring the need for reliable preclinical avatars to test regimens preclinically. In this study, we evaluated four combinations of three FDA-approved targeted agents (sunitinib, pazopanib, everolimus) in our PDOX platform. While these drugs have been studied individually in RCC PDOX models, no prior patient-derived orthotopic models have assessed sequential targeted therapy in vivo [34,44,45,46]. Our results revealed patient-specific optimal sequences (P→E most effective for KiCa-Pt58 tumor burden and metastases; S→E and P→E for KiCa-Pt118 growth inhibition), with corresponding reductions in proliferation, angiogenesis, and PD-L1 expression in responsive groups. Given the limited number of PDOX models (*n* = 2), these sequencing results should be interpreted as proof-of-principle evidence of the platform’s feasibility for individualized therapy exploration, rather than as generalizable treatment recommendations for aRCC. Both models showed baseline PD-L1 positivity that decreased significantly with effective regimens. These findings support future investigation of rational TKI/mTOR inhibitor plus immune checkpoint blockade combinations, ideally in humanized or immunocompetent model systems.

These findings position RCC PDOX avatars within a broader precision-medicine framework aimed at individualized therapy selection for advanced RCC. By recapitulating interpatient heterogeneity, metastatic behavior, and therapeutic responses in vivo, the platform provides a functional approach for evaluating adaptive treatment strategies. Recent advances in RCC precision oncology increasingly emphasize integration of molecular profiling, longitudinal biomarker monitoring, and standardized patient stratification to optimize therapeutic decision-making [47]. In parallel, complementary ex vivo platforms such as patient-derived organoids and functional drug-screening systems have emerged as promising tools for rapid assessment of individualized therapeutic sensitivity [48]. Compared with ex vivo systems, orthotopic PDOX avatars preserve tumor architecture, stromal interactions, angiogenesis, and metastatic progression within an in vivo microenvironment, thereby enabling dynamic evaluation of treatment response and resistance evolution. Together, these complementary approaches may contribute to integrated translational pipelines for personalized RCC therapy selection and sequencing.

Limitations of the current PDOX platform include the time and resources required for model establishment (though faster and more straightforward than traditional PDX approaches), which typically required 6–12 weeks from initial implantation to sufficient tumor material for orthotopic injection and treatment initiation in our hands. The full timeline from patient specimen receipt to completion of sequential therapy evaluation averaged approximately 10–14 weeks per model (including subcutaneous expansion, Luc/RFP transduction, enrichment, orthotopic engraftment, tumor establishment, and 4–8 weeks of treatment monitoring). While this exceeds the timeframe for guiding first-line therapy in rapidly progressing aRCC patients, the platform remains highly valuable for second-line or later sequencing decisions, resistance mechanism studies, and pre-clinical validation of personalized regimens in patients with slower disease kinetics or those eligible for clinical trial enrollment. Additional limitations include partial replacement of human stroma by murine elements during serial passage (mitigated by limiting the number of passages to minimize genomic drift and stromal substitution). Although histology, Ki67, and CD44 expression support preservation of key tumor features, this study did not include genomic or transcriptomic comparisons (e.g., WES or RNA-seq) of the parental tumors, subcutaneous xenografts, and orthotopic PDOX tumors. In addition, although therapeutic responses were associated with changes in proliferation, angiogenesis, and PD-L1 expression, the current study did not include comprehensive downstream pathway analyses (e.g., phospho-mTOR, VEGFR signaling intermediates, or broader transcriptomic profiling) to mechanistically dissect sequence-dependent treatment effects. Future studies in humanized orthotopic PDOX models should therefore prioritize targeted phospho-protein profiling to assess drug target engagement and adaptive signaling across each sequence. For everolimus-containing regimens, mTORC1 activity should be prioritized using p-S6 and p-4EBP1; for VEGFR-TKI regimens (sunitinib or pazopanib), angiogenic pathway engagement should be assessed using p-VEGFR2 [49,50,51]. Secondary readouts such as p-AKT and p-ERK1/2 may further inform sequence-dependent adaptation and potential cross-resistance, particularly when evaluated at standardized time points (baseline, early on-treatment, and immediately before/after the treatment switch) [49,52]. Such analyses will help confirm fidelity of RCC driver alterations across model stages and better define mechanisms underlying differential therapeutic responses.

Engraftment success was high for the two specimens used in this study (87% for KiCa-Pt58 and 77% for KiCa-Pt118 after optimization of cell dosing and stromal support), but we acknowledge that patient-derived RCC models can exhibit variable take rates in the literature (20–80% depending on tumor subtype, stage, and processing conditions). In our experience, sarcomatoid and metastatic tumors engrafted more readily than non-metastatic clear cell variants, consistent with prior reports. Only successfully established models were advanced to therapeutic testing, which may introduce selection bias toward more aggressive tumors but reflects the platform’s current utility for patients with higher-risk disease. Additionally, the use of NOD/SCID mice, which lack functional adaptive immunity, precludes direct evaluation of ICB therapies in this immunocompromised system. Consequently, the sequencing effects observed in this study primarily reflect tumor-intrinsic and stromal responses to targeted agents in the absence of adaptive immune pressure. However, the model faithfully preserves human tumor PD-L1 expression and antigen presentation, suggesting it could be adapted to humanized mouse platforms in future studies to enable combined targeted therapy plus ICB testing.

## 5. Conclusions

This luciferase-enabled RCC PDOX platform faithfully recapitulates patient-specific tumor biology, metastatic behavior, and therapeutic responses while enabling real-time evaluation of sequential targeted therapies. Differential outcomes between models highlight interpatient heterogeneity and underscore the potential of PDOX avatars to guide individualized treatment strategies in advanced RCC, including rational combinations with immune checkpoint blockade. These findings establish the proof of concept that patient-derived orthotopic xenograft models can evaluate individualized therapeutic strategies and resistance patterns and provide a foundation for future studies designed to test their predictive value for clinical treatment selection.

## Figures and Tables

**Table 1:** Patient information and pathology diagnoses for specimens used in this study.

	Age, Sex	Pathology Stage	Histologic Grade	Histologic Type	Tumor Size
KiCa-Pt58	56, male	pT3aN1M1 (adrenal gland)	Fuhrman grade 4	Sarcomatoid RCC with focal chromophobe differentiation	17.5 cm × 15 cm
KiCa-Pt118	71, male	pT3aNxM0	Fuhrman grade 4	Clear cell RCC with sarcomatoid component	8.0 cm × 6.5 cm

Patient characteristics and pathological features of the two renal cell carcinoma (RCC) cases used to establish the PDOX models. Staging is based on the American Joint Committee on Cancer/Tumor-Node-Metastasis (AJCC/TNM) system at the time of nephrectomy. Ki67 sarcomatoid differentiation is a known adverse prognostic feature associated with aggressive behavior and poor outcomes.

**Table 2:** Statistical significance of sequential targeted therapy regimens in PDOX models derived from KiCa-Pt58 and KiCa-Pt118.

	Treatment			
Endpoint	E→S	P→S	S→E	P→E
**KiCa-Pt58**				
Tumor weight	0.0018 (**)	ns	0.0337 (*)	0.0059 (**)
Lung BLI	ns	ns	0.0394 (*)	0.008 (**)
Ki67+	0.0141 (*)	ns	0.0384 (*)	0.0004 (***)
CD31+	ns	ns	0.0172 (*)	0.0306 (*)
PD-L1+	ns	ns	0.003 (**)	0.0116 (*)
**KiCa-Pt118**				
Tumor weight	ns	ns	0.002 (**)	0.0038 (**)
Ki67+	ns	ns	0.0006 (***)	0.0059 (**)
CD31+	ns	ns	0.0454 (*)	ns
PD-L1+	ns	ns	0.0116 (*)	ns

Summary of statistical significance for primary tumor burden (weight or % growth inhibition), lung metastatic burden (ex vivo BLI), and IHC biomarkers (Ki67+, CD31+, PD-L1+ area) across sequential treatment regimens in the KiCa-Pt58 and KiCa-Pt118 PDOX models. Effective sequences (with P→E showing the strongest effect in KiCa-Pt58 and both S→E and P→E being highly effective in KiCa-Pt118) showed significant reductions in tumor burden and biomarker expression compared to vehicle control. *, *p* < 0.05; **, *p* < 0.01; ***, *p* < 0.001; ns, not significant.

**Table 3:** PDOX model-specific summary of sequential targeted therapy performance.

PDOX Model	Defining Features	Preferred Sequence	Secondary Sequence(s)
KiCa-Pt58	Rapid engraftment, early progression, spontaneous lung metastases, fulminant patient course.	P→E	E→S; S→E
KiCa-Pt118	Delayed engraftment, stromal dependency, slower progression, no lung metastases, long recurrence-free patient course.	S→E	P→E

Sequence performance varied by PDOX model phenotype and growth behavior. Preferred and secondary sequences are summarized for translational interpretation. Detailed endpoint-level statistical results are provided in Table 2. E, everolimus; P, pazopanib; S, sunitinib.

## Data Availability

The data supporting the findings of this study are available within the article and its Supplementary Materials. Additional data may be available from the corresponding author upon reasonable request, subject to ethical and institutional restrictions related to patient-derived materials.

## Supplementary Materials

The following supporting information can be downloaded at https://www.mdpi.com/article/10.3390/cancers18101615/s1. Figure S1. Comparison of tumor growth kinetics between Luc/RFP-tagged and non-tagged renal cell carcinoma tumors in a subcutaneous model. Tumor volume (length × width × height in mm^3^) was measured in KiCa-Pt431-LucRFP (tagged) and parental non-tagged KiCa-Pt431 tumors. Although measurements were performed on slightly different schedules, the non-tagged tumors generally showed faster progression in the later stages. Data suggest that Luc/RFP transduction imposes only a modest growth disadvantage.

## Abbreviations

The following abbreviations are used in this manuscript:

aRCC	advanced renal cell carcinoma
BLI	bioluminescence image
CD31	cluster of differentiation 31
CD44	cluster of differentiation 44
E	everolimus
H&E	hematoxylin and eosin
FACS	fluorescence-activated cell sorting
FDA	Food and Drug Administration
ICB	immune checkpoint blockade
IACUC	institutional animal care and use committee
IHC	immunohistochemistry
IK	intra-kidney sub-capsular
IP	intraperitoneal
Ki-67	marker of proliferation Ki-67
KiCa-Pt	kidney cancer patient
Luc/RFP	luciferase/red fluorescent protein
mTOR	mammalian target of rapamycin
MRI	magnetic resonance imaging
NOD/SCID	non-obese diabetic/severe combined immunodeficient
OS	overall survival
P	pazopanib
PD-1	programmed death receptor-1
PD-L1	programmed death-ligand 1
PDOX	patient-derived orthotopic xenograft
PET-CT	positron emission tomography/computed tomography
PFS	progression-free survival
RCC	renal cell carcinoma
S	sunitinib
SEER	Surveillance, Epidemiology, and End Results Program
TKI	multi-tyrosine kinase inhibitor
VEGF	vascular endothelial growth factor
