# Supplementary figures and images for: Optimizing Sequential Targeted Therapies in Advanced Renal Cell Carcinoma Using Patient-Derived Orthotopic Xenograft Mouse Avatars
Source: Cancers (Basel). 2026 May 16;18(10):1615. doi: 10.3390/cancers18101615 (PMC13204348; doi:10.3390/cancers18101615)

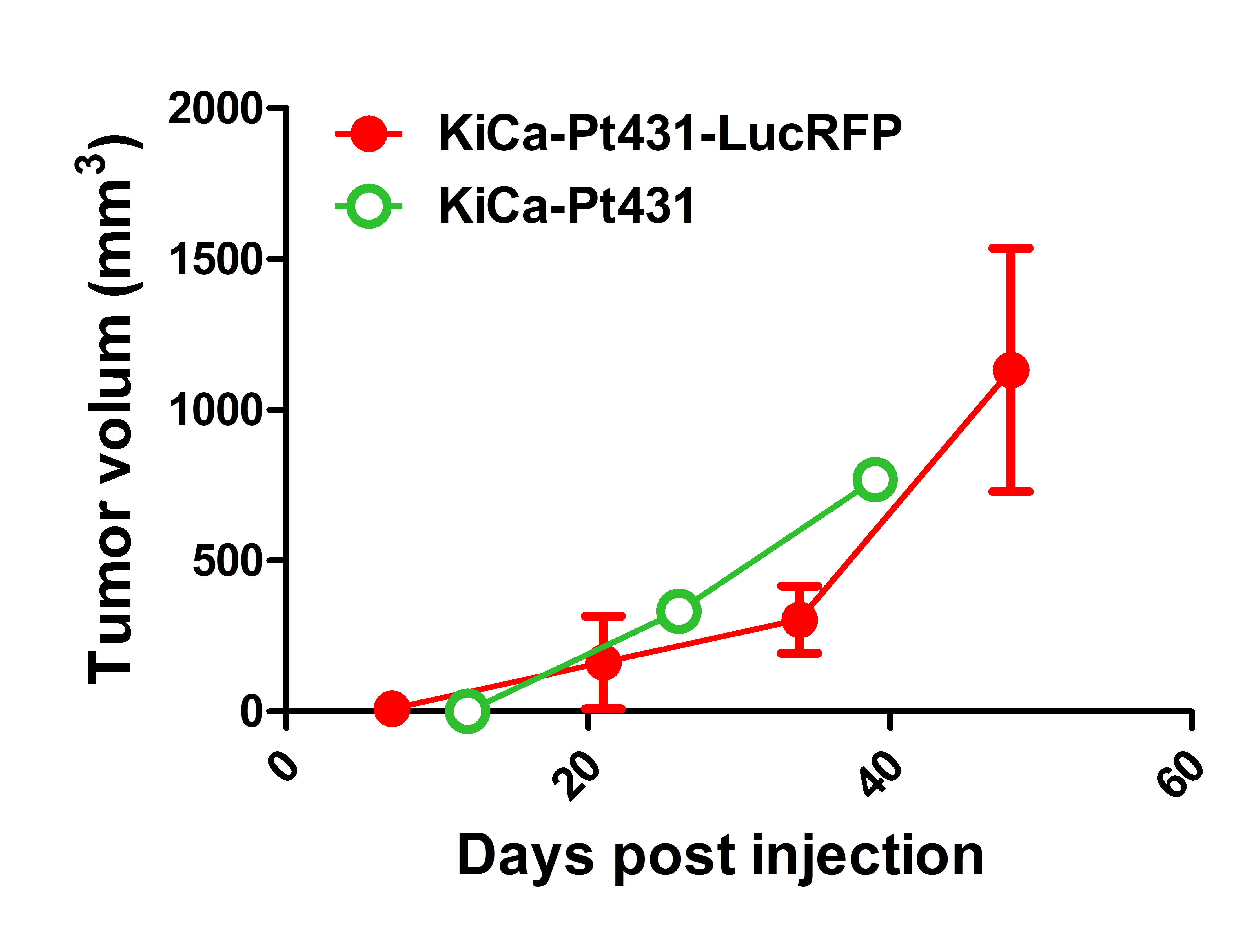

Supplement: Supplementary file 1 [file cancers-18-01615-s001.zip › cancers-4283928-supplementary.tif]
